# Clinical Timing-Sequence Warning Models for Serious Bacterial Infections in Adults Based on Machine Learning: Retrospective Study

**DOI:** 10.2196/45515

**Published:** 2023-12-18

**Authors:** Jian Liu, Jia Chen, Yongquan Dong, Yan Lou, Yu Tian, Huiyao Sun, Yuqing Jin, Jingsong Li, Yunqing Qiu

**Affiliations:** 1 Department of Intensive Care Unit the First Affiliated Hospital, College of Medicine, Zhejiang University Hangzhou China; 2 Research Center for Healthcare Data Science Zhejiang Laboratory Hangzhou China; 3 Department of Respiratory Disease Yinzhou Second Hospital Ningbo China; 4 Zhejiang Provincial Key Laboratory for Drug Clinical Research and Evaluation,Department of Clinical Pharmacy the First Affiliated Hospital, College of Medicine, Zhejiang University Hangzhou China; 5 Engineering Research Center of Electronic Medical Record and Intelligent Expert System, Ministry of Education, College of Biomedical Engineering and Instrument Science Zhejiang University Hangzhou China; 6 Research Center for Healthcare Data Science, Zhejiang Laboratory Engineering Research Center of Electronic Medical Record and Intelligent Expert System, Ministry of Education, College of Biomedical Engineering and Instrument Science, Zhejiang University Hangzhou China; 7 State Key Laboratory for Diagnosis and Treatment of Infectious Diseases, Zhejiang Provincial Key Laboratory for Drug Clinical Research and Evaluation the First Affiliated Hospital, College of Medicine, Zhejiang University Hangzhou China

**Keywords:** clinical timing-sequence warning models, machine learning, serious bacterial infection, nomogram

## Abstract

**Background:**

Serious bacterial infections (SBIs) are linked to unplanned hospital admissions and a high mortality rate. The early identification of SBIs is crucial in clinical practice.

**Objective:**

This study aims to establish and validate clinically applicable models designed to identify SBIs in patients with infective fever.

**Methods:**

Clinical data from 945 patients with infective fever, encompassing demographic and laboratory indicators, were retrospectively collected from a 2200-bed teaching hospital between January 2013 and December 2020. The data were randomly divided into training and test sets at a ratio of 7:3. Various machine learning (ML) algorithms, including Boruta, Lasso (least absolute shrinkage and selection operator), and recursive feature elimination, were utilized for feature filtering. The selected features were subsequently used to construct models predicting SBIs using logistic regression (LR), random forest (RF), and extreme gradient boosting (XGBoost) with 5-fold cross-validation. Performance metrics, including the receiver operating characteristic (ROC) curve and area under the ROC curve (AUC), accuracy, sensitivity, and other relevant parameters, were used to assess model performance. Considering both model performance and clinical needs, 2 clinical timing-sequence warning models were ultimately confirmed using LR analysis. The corresponding predictive nomograms were then plotted for clinical use. Moreover, a physician, blinded to the study, collected additional data from the same center involving 164 patients during 2021. The nomograms developed in the study were then applied in clinical practice to further validate their clinical utility.

**Results:**

In total, 69.9% (661/945) of the patients developed SBIs. Age, hemoglobin, neutrophil-to-lymphocyte ratio, fibrinogen, and C-reactive protein levels were identified as important features by at least two ML algorithms. Considering the collection sequence of these indicators and clinical demands, 2 timing-sequence models predicting the SBI risk were constructed accordingly: the early admission model (model 1) and the model within 24 hours of admission (model 2). LR demonstrated better stability than RF and XGBoost in both models and performed the best in model 2, with an AUC, accuracy, and sensitivity of 0.780 (95% CI 0.720-841), 0.754 (95% CI 0.698-804), and 0.776 (95% CI 0.711-832), respectively. XGBoost had an advantage over LR in AUC (0.708, 95% CI 0.641-775 vs 0.686, 95% CI 0.617-754), while RF achieved better accuracy (0.729, 95% CI 0.673-780) and sensitivity (0.790, 95% CI 0.728-844) than the other 2 approaches in model 1. Two SBI-risk prediction nomograms were developed for clinical use based on LR, and they exhibited good performance with an accuracy of 0.707 and 0.750 and a sensitivity of 0.729 and 0.927 in clinical application.

**Conclusions:**

The clinical timing-sequence warning models demonstrated efficacy in predicting SBIs in patients suspected of having infective fever and in clinical application, suggesting good potential in clinical decision-making. Nevertheless, additional prospective and multicenter studies are necessary to further confirm their clinical utility.

## Introduction

The detection and management of infectious diseases continue to be a primary concern for individuals experiencing fever [1]. The prevalence of infectious diseases is notably elevated, especially in regions characterized by low and lower-middle income [2,3]. Severe infectious diseases have the potential to rapidly advance to conditions such as sepsis, septic shock, or, in extreme cases, fatalities. This exerts significant strain on health care services and results in a depletion of intensive care resources. Swiftly identifying and accurately diagnosing serious bacterial infections (SBIs) are imperative to promptly initiate suitable treatments. The established gold standard for diagnosing infections in clinical samples relies on a process involving incubation, isolation, and identification. However, this method has its drawbacks, including a notable incidence of false-positive and false-negative outcomes from bacterial cultures. Additionally, the procedure is time-consuming, typically requiring 2-3 days or even longer to yield a definitive result [4,5]. The management of SBIs demands prompt action due to the time-sensitive nature of the condition, which can rapidly deteriorate. Nonetheless, the inappropriate use of broad-spectrum antibiotics or combinations of antimicrobials carries various drawbacks, including heightened health care expenditures, adverse drug reactions, and an elevated risk of developing drug-resistant bacteria. Therefore, the timely detection of SBIs in febrile patients suspected of having an infectious origin is paramount. This approach holds the potential to save lives and encourage a swift response without resorting to antibiotic misuse.

In this digital era, electronic health records serve as an extensive repository of electronic data points encompassing a wide range of clinical information [6]. Machine learning (ML) techniques possess a distinctive capability to analyze vast data sets in a flexible and trainable manner, enabling them to comprehend the intricate relationships between variables [7]. Owing to their enhanced processing capabilities, a variety of ML and artificial intelligence (AI) techniques are extensively used for identifying risk factors for diseases in patients and providing assistance to clinicians. Nevertheless, a previous study [8] has indicated that diverse feature selection methods and classification techniques can yield differing performance outcomes. Therefore, determining the optimal ML methods is essential for ensuring stable and accurate predictions when applied in clinical settings.

Several clinical models utilizing ML have been developed and validated for SBIs in infants and young children [9-11]. Yiu et al [12] specifically created a multivariable model to forecast the risk of serious infection in patients with psoriasis. Nonetheless, the discriminative ability was found to be unsatisfactory, with a C-statistic of only 0.64. Rawson et al [13] developed and validated a model to predict the presence of bacterial infection within 72 hours. However, the prediction did not prove to be timely enough for SBIs, and no applicable tools for physicians are currently available for use in their clinical practice. Qu et al [14] conducted a study comparing the timing-sequence recovery effect on exercise-induced muscle damage at various time points, including 1, 24, 48, and 72 hours, shedding new light on the timely prediction of SBIs. Building on the insights from this research, our study aims to develop and validate clinical timing-sequence warning models for SBIs using multiple ML methods. The goal is to provide physicians with practical and clinically applicable tools for use in their busy clinical practice for patients suspected of having an infective fever.

## Methods

### Study Design and Data Preprocessing

**Figure 1:**
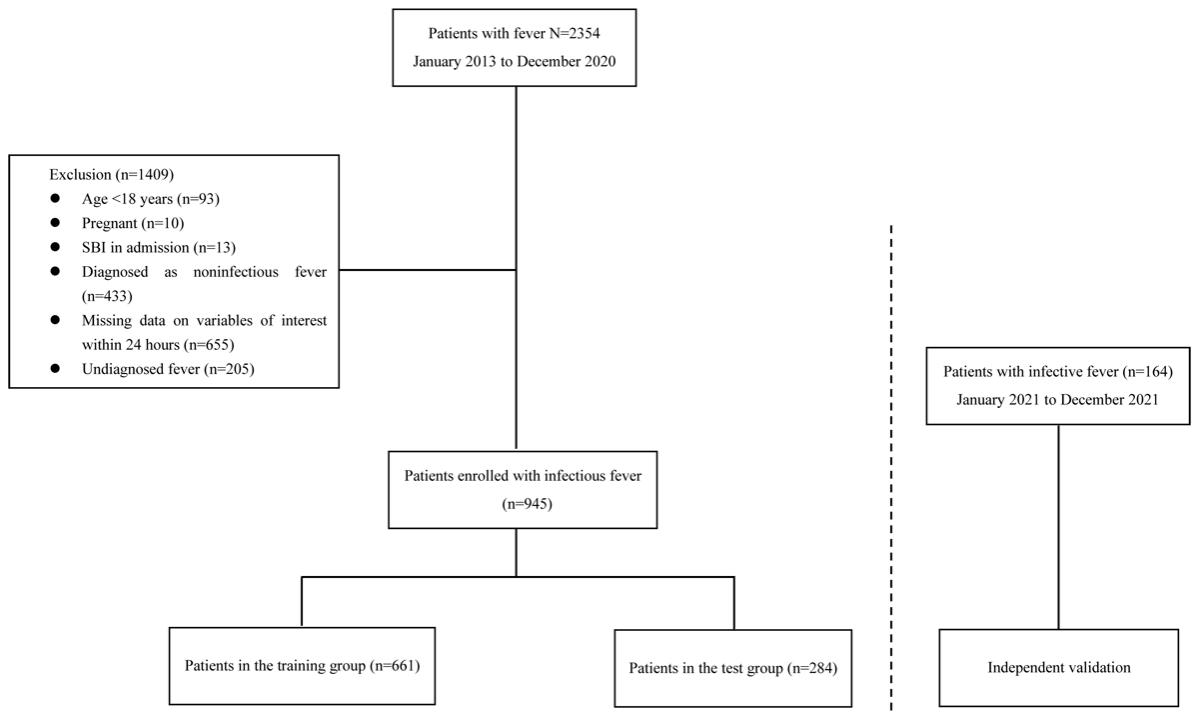
Patient enrollment flowchart. SBI: serious bacterial infection.

This retrospective observational cohort study was conducted at a 2200-bed teaching hospital, spanning from January 2013 to December 2020. An independent physician, blinded to the study, was invited to collect data from January to December 2021 within the same hospital. Patients were included in the study if they met the following criteria: (1) diagnosed with “fever of unknown origin” or presented with a chief complaint of fever persisting for several days, and (2) had a temperature of ≧37.5°C, indicative of fever. Exclusion criteria encompassed patients who met any of the following: (1) age <18 years, (2) pregnancy, (3) presence of an SBI upon admission, (4) noninfectious fever, (5) missing data on the variables of interest, and (6) undiagnosed fever. More specific details about the patient enrollment process are illustrated in Figure 1. The confirmation of SBI diagnosis involved discussions with the clinical research team and a review of the read code classification [15] and the International Classification of Diseases, 10th revision [16].

The variables of interest were obtained from the hospitalization records, encompassing demographic information such as sex, age, BMI, past medical history (hypertension, diabetes mellitus, heart disease, nephropathy, hepatitis B, and malignant tumors), as well as laboratory test results collected within the initial 24 hours of admission to the hospital. Outlier values, defined as falling below the 0.5th percentile or exceeding the 99.5th percentile, were adjusted to the values at the 0.5th percentile if lower or the 99.5th percentile if higher. Subsequently, these outliers were removed from the data set. Any other missing laboratory values were imputed using the K-nearest neighbors approach, replacing them with the mean of the respective feature from the 10 most similar samples in the training data. The data underwent normalization for each laboratory feature through transformations involving subtraction of the mean and division by the SD [17]. The research adhered to the CONSORT (Consolidated Standards of Reporting Trials) checklist [18] (Multimedia Appendix 1) and followed the “Guidelines for Developing and Reporting Machine Learning Predictive Models in Biomedical Research” [19].

### Ethics Considerations

Data retrieval received approval from the ethics committee of the First Affiliated Hospital, College of Medicine, Zhejiang University (ITT20220327B; date of approval: July 19, 2022). No potentially identifiable human images or data were included in the data collection, and the requirement for informed consent was thus waived.

### Data Partitioning and Statistical Analysis

The data were randomly classified into training and test sets at a ratio of 7:3, using equal proportion sampling. This was done to ensure that both the training and test sets encompassed approximately the same proportion of positive events (ie, SBIs). Descriptive statistics, including the IQR and frequency (proportion), were utilized to illustrate the distribution of the features of interest. The chi-square test (or the Fisher exact probability test) was applied for categorical variables, while the Wilcoxon rank sum test was used for continuous variables to assess the presence of differences in the distribution of clinical features between the 2 data sets. Differences between the SBI and non-SBI groups were assessed using either the Wilcoxon rank sum test or the chi-square test. Furthermore, the correlation between variables was gauged by calculating Pearson’s correlation coefficients and visualized in a heatmap. All statistical analyses and algorithms were executed using R, version 4.0.2 (R Foundation) statistical software.

### Feature Selection

Three popular algorithms—Boruta, Lasso (least absolute shrinkage and selection operator), and RFE (recursive feature elimination)—were utilized to screen important features in this study. This approach was adopted to prevent a preference bias that may arise from relying solely on 1 algorithm. To provide further detail, Boruta uses the random forest (RF) model as a classifier package algorithm [20]. Lasso operates on a linear regression model by selecting and compressing variables, effectively addressing the issue of overfitting [21]. By contrast, RFE selects features by recursively reducing the size of the examined feature set [22]. Features deemed important were those marked as such by at least two ML algorithms, a measure taken to enhance the simplicity and stability of the models.

### Model Construction

The chosen features were subsequently used to construct models predicting SBIs using logistic regression (LR), RF, and extreme gradient boosting (XGBoost) analyses, incorporating 5-fold cross-validation (CV). Considering the clinical timing of indicator acquisition (ie, basic characteristics and routine blood indices were assessed and obtained immediately after admission, while other serum biochemical indices were typically obtained 12-24 hours later) and the practical demands of clinical practice, 2 clinical timing-sequence warning models were developed. Specifically, these models were designed for 2 purposes: the prediction of SBI risk at early admission (model 1), incorporating basic characteristics plus routine blood indices, and a prediction within 24 hours of admission (model 2), utilizing basic characteristics plus biochemical indicators.

Performance metrics, including the receiver operating characteristic (ROC) curve and area under the ROC curve (AUC), accuracy, sensitivity, specificity, positive predictive value, negative predictive value, positive likelihood ratio (LR+), and negative likelihood ratio (LR–), were used. The optimal cutoff value was determined based on the Youden index.

### Construction and Evaluation of the Predictive Nomogram

Nomograms predicting the risk of SBIs were generated based on the best model, providing a convenient tool for physicians to assess SBI risk for individual patients in clinical practice. Independent data were utilized for further validation of the nomogram’s performance and clinical utility. Additionally, to enhance practical usability for health care professionals globally and enable open validation by peers, we have implemented the 2 nomogram tools online using DynNom [23,24]. Through this platform, the SBI risk score can be automatically generated and visually displayed.

## Results

### Patient Characteristics

In this study, a total of 945 patients clinically diagnosed with infective fever and possessing complete clinical data were included. Table 1 presents the demographic characteristics of these patients. Among them, data from 661 patients were utilized for training, while data from 284 patients were used for validation. The median age of the patients was 54 years (IQR 38-66 years), and 47.4% (448/945) were female. The most prevalent comorbidity among the patients was hypertension, affecting 27.9% (264/945), while other comorbidities such as diabetes mellitus, cardiopathy, and nephropathy were observed in 12.1% (114/945), 6.3% (60/945), and 5.5% (52/945) of patients, respectively. In the training cohort, consisting of 661 patients, 69% (456/661) were diagnosed with SBIs. The median age of the patients was 59 years, and among them, 226 were female. Further clinical characteristics of the patients with and without SBIs are detailed in Multimedia Appendix 2.

Regarding the serious infectious episodes, the top 3 clinical sites of infections were the lung (pneumonia; 224/945, 23.7%), the urinary tract (83/945, 8.8%), and the abdomen (78/945, 8.3%). Based on the clinical course and microbiological data, bacterial infections and viral infections were present in 717 patients (717/945, 75.9%) and 185 patients (185/945, 19.6%), respectively (Multimedia Appendix 3).

**Table 1:** Differences among patients in derivation and validation cohorts.

Differences between the cohorts			Overall		Training cohort		Test cohort		*P* value
Patients, n			945		661		284		
Female, n (%)			448 (47.4)		314 (47.5)		134 (47.2)		.98
Age (year), median (IQR)			54.00 (38.00-66.00)		54.00 (38.00-67.00)		56.00 (43.00-64.00)		.83
BMI (kg/m^2^), median (IQR)			22.04 (19.71-24.33)		22.05 (19.82-24.38)		21.88 (19.59-24.23)		.44
Ventricular rate (beats/minute), median (IQR)			90.00 (79.00-102.00)		90.00 (79.00-101.00)		92.00 (80.00-103.00)		.22
Respiratory rate (breaths/minute), median (IQR)			19.00 (18.00-20.00)		19.00 (18.00-20.00)		18.00 (18.00-20.00)		.29
Ear temperature (℃), median (IQR)			37.30 (36.90-37.90)		37.30 (36.90-37.90)		37.40 (36.90-38.00)		.80
Diastolic pressure (mm Hg), median (IQR)			71.00 (64.00-79.00)		71.00 (64.00-79.00)		71.00 (64.75-79.00)		.93
Systolic pressure (mm Hg), median (IQR)			115.00 (105.00-127.00)		116.00 (105.00-128.00)		115.00 (104.00-126.00)		.53
Hemoglobin (g/L), median (IQR)			117.00 (104.00-130.00)		116.00 (104.00-130.00)		118.00 (104.00-130.25)		.37
White blood cell (×10^9^/L), median (IQR)			6.60 (4.80-9.40)		6.50 (4.70-9.30)		6.80 (5.00-9.50)		.41
Platelet (×10^9^/L), median (IQR)			217.00 (152.00-289.00)		215.00 (149.00-282.00)		226.00 (159.50-309.50)		.12
Neutrophils proportion (%), median (IQR)			70.10 (58.80-79.30)		69.60 (57.40-78.80)		71.90 (60.55-80.32)		.03
Lymphocytes proportion (%), median IQR)			18.70 (11.60-28.40)		18.80 (12.10-28.70)		18.20 (10.78-27.25)		.07
Monocytes proportion (%), median (IQR)			8.10 (5.90-10.40)		8.20 (6.00-10.60)		7.60 (5.70-10.00)		.02
Eosinophils proportion (%), median (IQR)			0.80 (0.20-1.90)		0.80 (0.20-2.00)		0.75 (0.20-1.83)		.59
Basophils proportion (%), median (IQR)			0.30 (0.20-0.50)		0.30 (0.20-0.50)		0.30 (0.20-0.50)		.16
Neutrophil count (×10^9^/L), median (IQR)			4.20 (2.90-7.00)		4.20 (2.80-6.80)		4.35 (3.10-7.40)		.14
Lymphocyte count (×10^9^/L), median (IQR)			1.22 (0.80-1.75)		1.26 (0.80-1.77)		1.15 (0.78-1.69)		.16
Monocyte count (×10^9^/L), median (IQR)			0.52 (0.36-0.78)		0.53 (0.36-0.79)		0.51 (0.36-0.73)		.30
Eosinophil count (×10^9^/L), median (IQR)			0.05 (0.01-0.13)		0.05 (0.01-0.13)		0.05 (0.01-0.13)		.84
Basophil count (×10^9^/L), median (IQR)			0.02 (0.01-0.03)		0.02 (0.01-0.03)		0.02 (0.01-0.04)		.17
Blood glucose (mmol/L), median (IQR)			4.99 (4.41-5.82)		4.97 (4.41-5.78)		5.00 (4.42-6.05)		.41
Albumin (g/L), median (IQR)			35.30 (31.30-38.70)		35.50 (31.30-38.80)		34.95 (31.35-38.02)		.31
Creatinine (μmol/L), median (IQR)			67.00 (55.00-82.00)		66.00 (55.00-83.00)		68.50 (55.00-81.00)		.90
D-dimer (μg/L), median (IQR)			1276.00 (604.50-3008.00)		1311.50 (625.00-2914.75)		1163.00 (543.00-3331.00)		.65
Activated partial thromboplastin time (seconds), median (IQR)			30.30 (27.10-34.40)		30.20 (27.28-34.30)		30.30 (26.80-34.60)		.81
Fibrinogen (g/L), median (IQR)			4.38 (3.22-5.44)		4.22 (3.10-5.42)		4.57 (3.43-5.53)		.04
C-reactive protein (mg/L), median (IQR)			36.30 (12.07-81.71)		34.70 (10.70-77.40)		44.10 (14.00-91.45)		.04
Procalcitonin (ng/mL), median (IQR)			0.15 (0.06-0.40)		0.14 (0.06-0.36)		0.17 (0.08-0.53)		.09
Neutrophil-to-lymphocyte ratio, median IQR			3.75 (2.09-6.72)		3.69 (2.03-6.39)		4.01 (2.25-7.56)		.05
Serious bacterial infection, n (%)			661 (69.9)		456 (69.0)		205 (72.2)		.36
**Comorbidities, n (%)**									.76
	0	542 (57.4)		375 (56.7)		167 (58.8)			
	1	241 (25.5)		169 (25.6)		72 (25.4)			
	2+	162 (17.1)		117 (17.7)		45 (15.8)			
	Hypertension, n (%)	264 (27.9)		191 (28.9)		73 (25.7)		.36	
	Diabetes, n (%)	114 (12.1)		74 (11.2)		40 (14.1)		.25	
	Cardiopathy, n (%)	60 (6.3)		40 (6.1)		20 (7.0)		.67	
	Nephropathy, n (%)	52 (5.5)		40 (6.1)		12 (4.2)		.33	
	Hepatitis B, n (%)	41 (4.3)		29 (4.4)		12 (4.2)		>.99	
	Malignancy, n (%)	35 (3.7)		26 (3.9)		9 (3.2)		.70	

### Characteristics of the Selected Features

**Figure 2:**
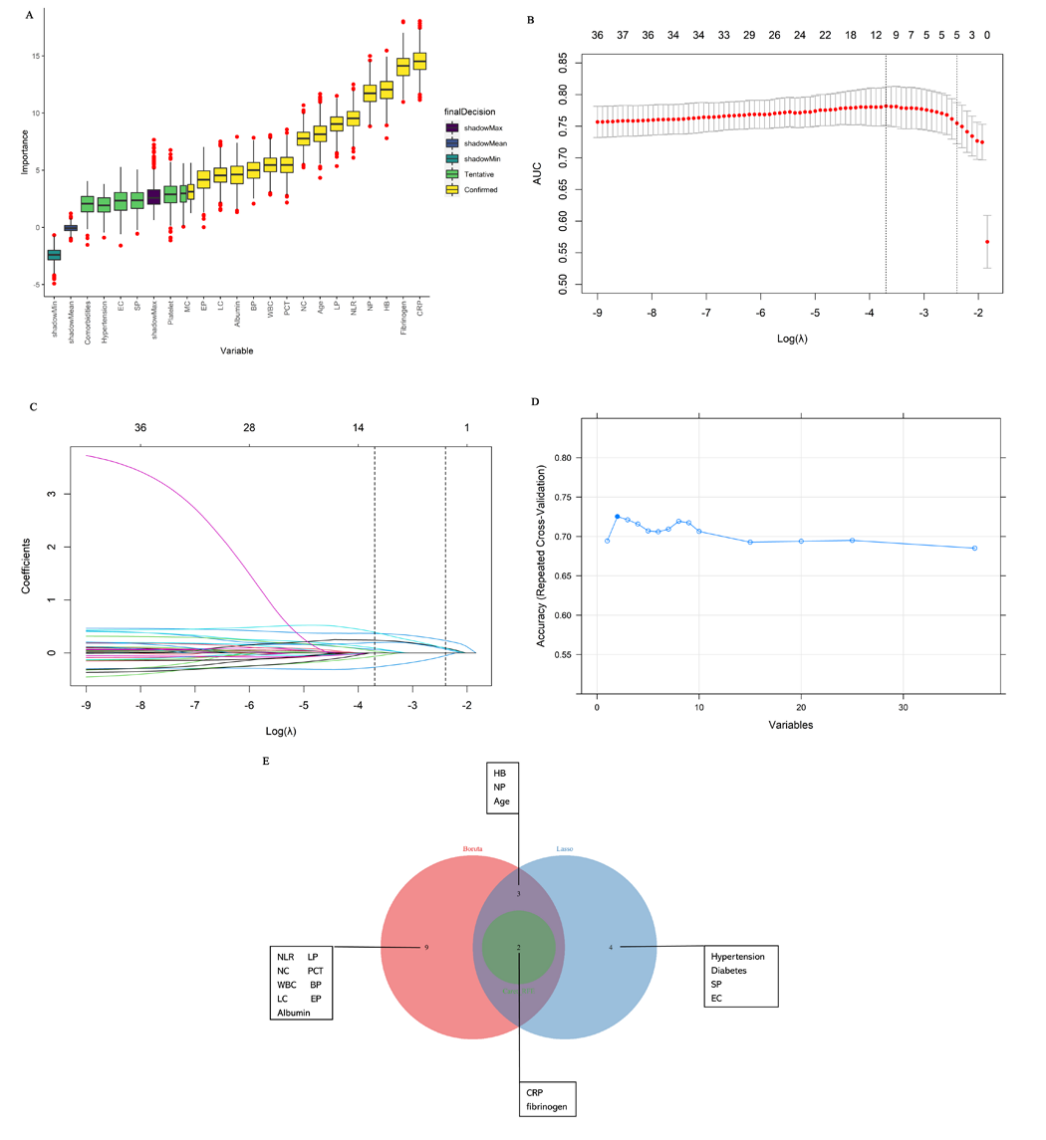
Process of feature selection. (A) The rank of feature importance sorted by Boruta; 14 features were marked as significant. (B and C) The best parameter (lambda) and best number of indicators (ie, 9) for the best performance in the Lasso algorithm. (D) RFE included 2 features to obtain the top accuracy. (E) The union of all the features selected by the Boruta, Lasso, and RFE algorithms. BP: basophils proportion; CRP: C-reactive protein; EC: eosinophil count; EP: eosinophils proportion; HB: hemoglobin; Lasso: least absolute shrinkage and selection operator; LC: lymphocyte count; LP: lymphocytes proportion; MC: monocyte count; NC: neutrophil count; NLR: neutrophil-to-lymphocyte ratio; NP: neutrophils proportion; PCT: procalcitonin; RFE: recursive feature elimination; SP: systolic pressure; WBC: white blood cell.

A total of 37 indicators, spanning demographic variables, clinical characteristics, vital signs, and blood biochemical indicators, were screened and compared. Notably, 14, 9, and 2 features were identified as important by the Boruta, Lasso, and RFE algorithms, respectively (Figure 2A-2D). A total of 18 independent features were recognized, with nearly all demonstrating significant differences (*P*<.05) between the SBI and non-SBI groups (Multimedia Appendix 2). However, notable collinearity was observed among these indicators, as depicted in Multimedia Appendix 4. To enhance the simplicity and stability of the models, features identified as important by at least two ML algorithms were chosen for model construction. These included age, neutrophil proportion (NP), hemoglobin, C-reactive protein (CRP), and fibrinogen levels (Figure 2E). Additionally, given that the neutrophil-to-lymphocyte ratio (NLR) simultaneously reflects lymphocyte proportions and NPs and is commonly used in clinical practice, it was used as a replacement for NP.

### Model Performance Result

Considering the sequence of collection for the chosen indicators and the clinical requirements, 2 timing-sequence models for predicting SBI risk were formulated. The early admission model (model 1) incorporated age, hemoglobin level, and NLR, while the model within 24 hours of admission (model 2) included age, hemoglobin, NLR, fibrinogen, and CRP levels. LR exhibited greater stability compared with RF and XGBoost in the 5-fold CV of both models. For model 1, LR achieved AUCs of 0.749 (5-fold CV training set) and 0.744 (5-fold CV validation set), while for model 2, LR showed AUCs of 0.806 (5-fold CV training set) and 0.807 (5-fold CV validation set; see panels A and B in Multimedia Appendix 5). Moreover, LR outperformed the other 2 algorithms in model 2, with an AUC, accuracy, and sensitivity of 0.780 (95% CI 0.720-841), 0.754 (95% CI 0.698-804), and 0.776 (95% CI 0.711-832), respectively, as presented in Tables 2 and 3. In model 1, XGBoost demonstrated an advantage over LR in terms of AUC (0.708, 95% CI 0.641-775 vs 0.686, 95% CI 0.617-754), while RF achieved superior accuracy (0.729, 95% CI 0.673-780) and sensitivity (0.790, 95% CI 0.728-844) compared with the other 2 algorithms (Tables 2 and 3).

**Table 2:** Model characteristics among the training and test sets: the early admission assessment model.

Cohort and model			AUC^a^ (95% CI)		Accuracy (95% CI)		Sensitivity (95% CI)		Specificity (95% CI)		PPV^b^ (95% CI)		NPV^c^ (95% CI)		LR (+)^d^ (95% CI)		LR (–)^e^ (95% CI)
**Training set**																	
	LR^f^	0.756 (0.715-0.796)		0.684 (0.647-0.719)		0.638 (0.592-0.682)		0.785 (0.723-0.840)		0.869 (0.828-0.903)		0.494 (0.438-0.550)		2.973 (2.268-3.898)		0.461 (0.400-0.531)	
	RF^g^	0.723 (0.679-0.766)		0.731 (0.695-0.764)		0.811 (0.772-0.846)		0.551 (0.480-0.621)		0.801 (0.761-0.836)		0.568 (0.496-0.638)		1.808 (1.544-2.118)		0.342 (0.273-0.429)	
	XGBoost^h^	0.796 (0.758-0.833)		0.770 (0.736-0.802)		0.805 (0.765-0.840)		0.693 (0.625-0.755)		0.853 (0.816-0.886)		0.615 (0.549-0.678)		2.619 (2.122-3.232)		0.282 (0.229-0.347)	
**Test set**																	
	LR	0.686 (0.617-0.754)		0.634 (0.575-0.690)		0.605 (0.534-0.672)		0.709 (0.596-0.806)		0.844 (0.775-0.898)		0.409 (0.326-0.496)		2.078 (1.447-2.982)		0.557 (0.447-0.695)	
	RF	0.685 (0.612-0.759)		0.729 (0.673-0.780)		0.790 (0.728-0.844)		0.570 (0.453-0.681)		0.827 (0.766-0.877)		0.511 (0.402-0.619)		1.836 (1.411-2.389)		0.368 (0.265-0.511)	
	XGBoost	0.708 (0.641-0.775)		0.676 (0.618-0.730)		0.663 (0.594-0.728)		0.709 (0.596-0.806)		0.855 (0.791-0.906)		0.448 (0.359-0.540)		2.279 (1.594-3.258)		0.475 (0.374-0.603)	

^a^AUC: area under the ROC curve.

^b^PPV: positive predictive value.

^c^NPV: negative predictive value.

^d^LR (+): positive likelihood ratio.

^e^LR (–): negative likelihood ratio.

^f^LR: logistic regression.

^g^RF: random forest.

^h^XGBoost: extreme gradient boosting.

**Table 3:** Model characteristics among the training and test sets: the risk assessment model within 24 hours of admission.

Cohort and model		AUC^a^ (95% CI)	Accuracy (95% CI)	Sensitivity (95% CI)	Specificity (95% CI)	PPV^b^ (95% CI)	NPV^c^ (95% CI)	LR (+)^d^ (95% CI)	LR (–)^e^ (95% CI)
**Training set**									
	LR^f^	0.807 (0.771-0.844)	0.777 (0.742-0.808)	0.839 (0.801-0.872)	0.640 (0.569-0.706)	0.837 (0.799-0.870)	0.643 (0.572-0.710)	2.330 (1.928-2.815)	0.252 (0.199-0.320)
	RF^g^	0.809 (0.772-0.846)	0.767 (0.732-0.799)	0.775 (0.733-0.813)	0.750 (0.684-0.808)	0.872 (0.835-0.904)	0.602 (0.539-0.664)	3.100 (2.426-3.962)	0.300 (0.248-0.363)
	XGBoost^h^	0.846 (0.812-0.879)	0.748 (0.713-0.782)	0.698 (0.652-0.740)	0.860 (0.804-0.905)	0.916 (0.881-0.944)	0.564 (0.506-0.620)	4.984 (3.516-7.065)	0.351 (0.302-0.409)
**Test set**									
	LR	0.780 (0.720-0.841)	0.754 (0.698-0.804)	0.776 (0.711-0.832)	0.697 (0.581-0.798)	0.869 (0.809-0.915)	0.546 (0.442-0.648)	2.563 (1.807-3.635)	0.322 (0.239-0.434)
	RF	0.759 (0.691-0.826)	0.706 (0.648-0.759)	0.694 (0.624-0.758)	0.737 (0.623-0.831)	0.872 (0.809-0.920)	0.483 (0.389-0.577)	2.637 (1.790-3.885)	0.415 (0.324-0.533)
	XGBoost	0.780 (0.717-0.843)	0.717 (0.659-0.770)	0.689 (0.619-0.753)	0.789 (0.681-0.875)	0.894 (0.834-0.938)	0.496 (0.404-0.588)	3.272 (2.096-5.108)	0.394 (0.311-0.500)

^a^AUC: area under the ROC curve.

^b^PPV: positive predictive value.

^c^NPV: negative predictive value.

^d^LR (+): positive likelihood ratio.

^e^LR (–): negative likelihood ratio.

^f^LR: logistic regression.

^g^RF: random forest.

^h^XGBoost: extreme gradient boosting.

### Evaluation of the Predictive Nomogram

**Figure 3:**
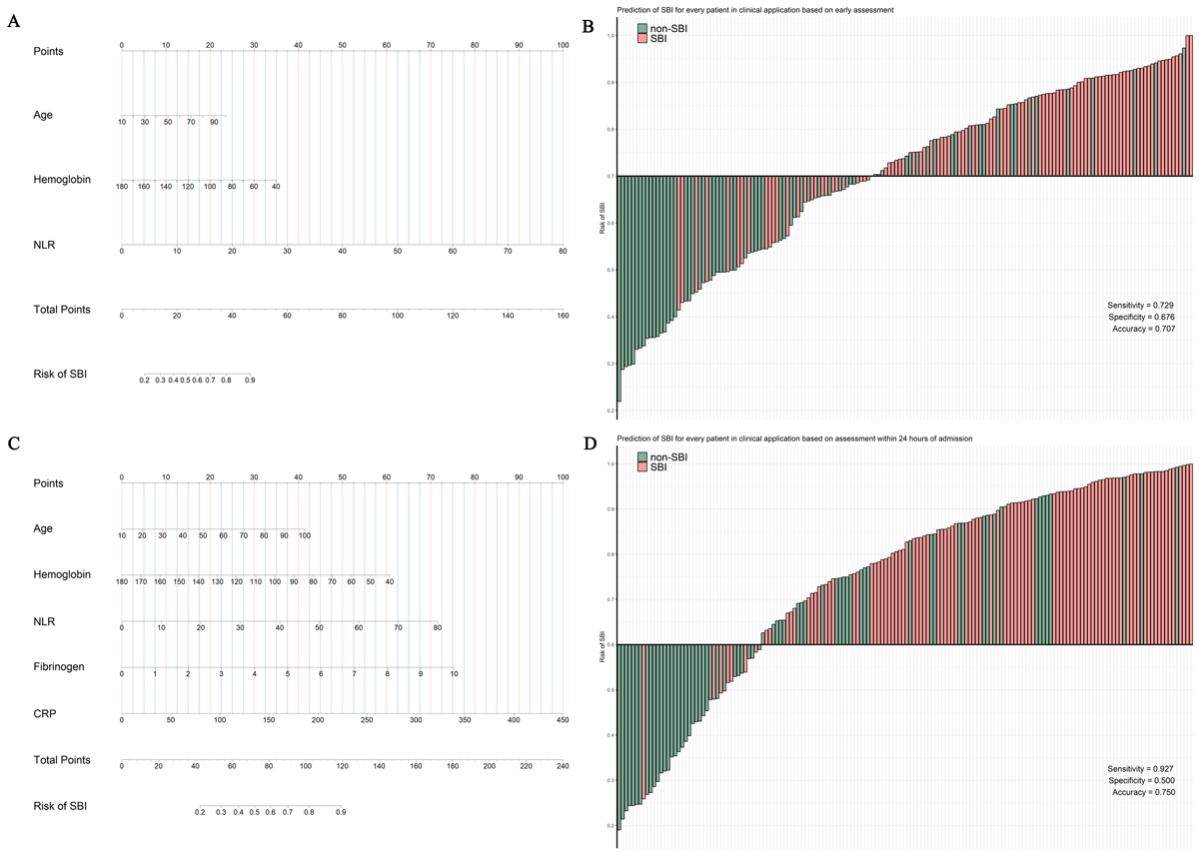
The predictive nomograms and their evaluation in clinical application. The predictive nomogram was developed to assess the SBI risk using (A) the early admission model and (C) the model within 24 hours of admission. The evaluation of the SBI risk for 164 patients in clinical application based on (B) the early assessment and (D) the assessment within 24 hours of admission. The height of each bar represents the SBI risk score for each patient, and the filling color indicates their actual outcomes. SBI: serious bacterial infection.

Two SBI-predicting nomograms based on LR, offering a succinct SBI risk score for each patient, were developed for models 1 and 2 (Figure 3A and 3C). To assess the clinical utility of the predictive nomograms, data on 164 patients diagnosed with infective fever and possessing complete prediction indicators (ie, age, hemoglobin level, lymphocyte count, neutrophil count, fibrinogen level, and CRP level) collected in 2021 were included. The early predictive nomogram exhibited good accuracy and sensitivity, with accuracy at 0.707 and sensitivity at 0.729. In the assessment of the model within 24 hours of admission, high sensitivity and good accuracy were observed, with sensitivity at 0.927 and accuracy at 0.750 (Figure 3B and 3D). These results demonstrate practical utility in clinical practice for distinguishing SBIs from non-SBIs. Two dynamic nomogram tools are deployed online [23,24].

## Discussion

### Principal Findings

Given the advancement of AI and the growing need for extensive data in clinical medicine, the integration of AI techniques into clinical practice and research holds significant importance. This retrospective study focused on the development of 2 timing-sequence SBI-risk models for adults with infectious fever. Leveraging multiple ML algorithms, these models were designed with a minimal set of 3 and 5 features, aiming to achieve accurate and stable predictions. The nomograms developed and validated for physicians provide a straightforward and convenient way to assess the risk of SBI for each patient in clinical practice. This suggests their promising potential in clinical decision-making, aiming to minimize unnecessary hospitalizations and the empirical use of antibiotics. As far as our knowledge extends, this study is the first to utilize timing-sequence models for detecting SBI in adults with suspected infectious fever, and their validation in clinical applications adds a novel dimension to the existing body of research.

The utilization of various feature screening methods comes with its set of advantages and disadvantages. In this study, we opted for a comprehensive approach by combining multiple feature screening methods with clinician advice for the final confirmation of features. The features selected by multiple ML algorithms, namely, age, hemoglobin levels, CRP levels, fibrinogen levels, and the NLR, align with the consensus in the current management of SBIs. This alignment enhances the reliability and interpretability of the models we constructed.

Indeed, aging has been associated with a decrease in the efficiency of bacterial killing by neutrophils under ex vivo conditions [25]. The reduced ability of older adults to mount an adequate immune response makes them more susceptible to bacterial infections compared with their younger counterparts [26].

The SBI group exhibited lower hemoglobin levels and higher CRP levels, aligning with findings from prior studies [27,28]. The inflammatory response to an infection, characterized by the secretion of interleukin-6 (IL-6) in significant amounts, could be a primary factor contributing to anemia [29,30]. IL-6 plays a role in upregulating hepcidin transcription, inhibiting the transport of intracellularly stored iron from macrophages and hepatocytes into the plasma, and reducing intestinal iron uptake, resulting in low levels of iron in the plasma [31]. The mechanism, initiated within hours of infection, restricts the availability of plasma iron required for erythroblast proliferation, consequently inhibiting erythropoiesis [31]. CRP is an acute-phase reactive protein released in significant amounts from the liver upon stimulation with IL-6 and other cytokines [32]. In the presence of infection, CRP plays dual roles by exerting both proinflammatory and anti-inflammatory effects. Its functions are mediating the elimination of pathogens and inhibiting the interaction between white blood cells and endothelial cells. Consequently, CRP is frequently used for the early diagnosis of infectious diseases. However, the diagnostic value of CRP alone for bacterial infections is considered only moderate. Indeed, elevated CRP levels are observed not only in pediatric patients infected with adenovirus in the absence of a secondary bacterial infection but also in patients with bacterial infections. This suggests that both viruses and bacteria can trigger an inflammatory host response [33].

Fibrinogen possesses an intrinsic ability to combat invading bacterial pathogens [34]. In a recent study, wild-type mice demonstrated rapid and efficient clearance of *Staphylococcus aureus*, while mice lacking fibrinogen (Fbg^–^) failed to clear the microbes. This disparity in the early clearance of bacteria resulted in all wild-type mice surviving the infection even after 2 weeks, whereas all Fbg^–^ mice succumbed within 24 hours [35]. Fibrinogen serves as an antimicrobial host defense protein through various mechanisms, including (1) establishing a physical barrier at the air–liquid interface, (2) entrapping bacteria in fibrinogen or fibrin networks, and (3) facilitating the recruitment and activation of host immune cells [34].

The sensitivity of the NLR in the diagnosis of bacteremia, infection, and sepsis has been validated in numerous studies [36-38]. Aird [39] reported that the hematologic system plays a primary role in sepsis pathogenesis. In systemic infection and sepsis, all blood cells undergo activation, leading to significant changes in their functions, counts, receptor expression, humoral substances, and the secretion of various signaling molecules. A complete blood count can offer a wealth of valuable information, including parameters such as the NLR.

The clinical presentation of SBIs is often variable and nonspecific [40]. Therefore, the application of ML to assess SBI risk is feasible, given its capability to identify associations not previously considered among a large number of variables. Moreover, the potential of ML tends to increase with larger data sets. These 3 ML algorithms are extensively used in constructing clinical prediction models and consistently demonstrate excellent performance. Given the variation in information, specifically blood indicators, obtained at admission among patients, we devised 2 sequential models to enhance clinical applicability. Both models demonstrated commendable performance in early and late evaluations, aiding in the early warning of SBIs. In the early model (model 1), the RF model yielded the best results, achieving the highest accuracy scores (0.731 for the training set and 0.729 for the test set). RF is an ensemble learning method known for its effectiveness in various classification and regression tasks [41]. It operates as a classifier that leverages multiple trees for training and predicting outcomes. Notably, RF exhibits high accuracy and is adept at balancing errors when analyzing data sets with unbalanced classifications [42]. In the later model (model 2), the LR model demonstrated an excellent accuracy score, achieving 0.777 for the training set and 0.754 for the test set. LR is a classic predictive analysis algorithm rooted in the concept of probability. It utilizes a sigmoid function to derive the predicted output for classification [10]. To visualize and quantify the LR results, a nomogram was constructed, with regression coefficients standardized and expressed as risk scores on a number line. The nomogram serves as a highly practical visual tool for constructing clinical models, offering great convenience for physicians in busy clinical practice. Our nomograms were rigorously tested to demonstrate their capability to sensitively and accurately identify patients at a high risk of SBIs. This aspect is critical in clinical practice, providing physicians with timely alerts to take proactive measures and avoid the development of severe conditions. Additionally, we deployed online dynamic nomogram tools, making model parameters publicly accessible on the same website. This setup enables health care professionals and peers to openly validate the clinical utility of the model, further enhancing its practical usage.

### Strengths

The strength of this study lies in the meticulous process of feature capture, ensuring that the selected covariates are highly relevant to the risk of SBIs in a clinical setting. This approach facilitates the construction of concise and stable models with clinical interpretation. We anticipate that the findings of our study could serve as a valuable reference for the application of ML models in various health care systems. Second, our study introduced and developed clinical timing-sequence warning models, representing an innovative approach adaptable to complex clinical situations. Third, we constructed and validated nomograms to visualize and quantify the risk of SBI for each patient, providing a practical tool for clinical application.

### Limitations

Our study has several limitations. The retrospective nature of the study may introduce bias in the analysis of the results. Additionally, the predictive model was generated using 1 data set and tested on another, which could impact the generalizability of the findings. While we believe that our model has undergone validation, it is crucial to apply it in other health institutions to assess its generalizability and test its applicability in clinical practice. Second, due to the retrospective design of the study, data were solely collected from past medical records. Recent laboratory tests that could be linked to SBIs, such as lactate levels, were not included as they were not tested.

### Conclusions

Early prediction of SBIs in adults is a crucial clinical requirement. Our research has the potential to assist clinicians in identifying SBIs in adults soon after onset, facilitating early clinical monitoring and treatment while avoiding unnecessary antibiotic use. In the future, additional prospective and multicenter studies are essential to further confirm the clinical utility of these models.

## Appendix

Multimedia Appendix 1 CONSORT (Consolidated Standards of Reporting Trials) checklist.

Multimedia Appendix 2 Differences among patients with and without SBI in the training cohort. SBI: serious bacterial infection.

Multimedia Appendix 3 Etiology distribution of 945 patients with fever.

Multimedia Appendix 4 Heatmap of the selected factors by Boruta, Lasso and RFE. Lasso: least absolute shrinkage and selection operator; RFE: recursive feature elimination.

Multimedia Appendix 5 The AUCs of the 2 models with 5-fold cross-validation. (A) Model 1: The distribution of AUCs obtained using the 3 ML algorithms in the 5-fold CV (a) training set and (c) validation set; the ROCs with 5-fold CV for the (b) training set and (d) validation set. (B) Model 2: The distribution of AUCs obtained using the 3 ML algorithms with 5-fold CV for the (a) training set and (c) validation set; the ROCs with 5-fold CV for the (b) training set and (d) validation set. AUC: AUC: area under the ROC curve; ROC: receiver operating characteristic; CV: coefficient of variation; ML: machine learning.

## Abbreviations

AI: artificial intelligence
AUC: area under the ROC curve
CONSORT: Consolidated Standards of Reporting Trials
CRP: C-reactive protein
CV: cross-validation
IL: interleukin
Lasso: least absolute shrinkage and selection operator
LR: logistic regression
ML: machine learning
NLR: neutrophil-to-lymphocyte ratio
NP: neutrophil proportion
RF: random forest
RFE: recursive feature elimination
ROC: receiver operating characteristic
SBI: serious bacterial infection
XGBoost: extreme gradient boosting

## Data Availability

Data archiving is not mandated but data will be made available upon reasonable request.
