# Supplementary material for: Clinical Timing-Sequence Warning Models for Serious Bacterial Infections in Adults Based on Machine Learning: Retrospective Study
Source: J Med Internet Res. 2023 Dec 18;25:e45515. doi: 10.2196/45515 (PMC10758945; doi:10.2196/45515)
Supplement: Multimedia Appendix 2 [file jmir_v25i1e45515_app2.doc]

| **Supplementary Table1 Differences Among Patients With and Without SBI in the Train Cohort** | | | |
| --- | --- | --- | --- |
| **Variable** | **Non-SBI** | **SBI** | ***P*** |
| Patients | 205 | 456 |  |
| Female,n, % | 88(42.9) | 226(49.6) | .14 |
| Age,year,median(IQR) | 42.00(30.00, 58.00) | 59.00(44.00, 69.00) | <0.001 |
| BMI,kg/m2,median(IQR) | 22.27(19.98, 24.54) | 22.03(19.67, 24.25) | .19 |
| VR,beats/min,median(IQR) | 90.0079.00, 100.00) | 90.00(79.00, 102.00) | .37 |
| RR,beats/min,median(IQR) | 19.00(18.00, 20.00) | 19.00(18.00, 20.00) | .45 |
| ET,℃, median(IQR) | 37.30(37.00, 37.90) | 37.30(36.90, 37.90) | .50 |
| DP,mmHg,median( IQR) | 72.00(64.00, 81.00) | 71.00(63.75, 78.00) | .20 |
| SP, mmHg,median( IQR) | 114.00(105.00, 125.00) | 116.00(105.00, 129.25) | .08 |
| HB, g/l, median(IQR) | 127.00(112.00, 142.00) | 112.00(101.00, 124.00) | <0.001 |
| WBC,10e9/L,median(IQR) | 5.90(4.00, 7.60) | 7.00(5.10, 10.20) | <0.001 |
| Platelet,10e9/L,median(IQR) | 188.00(126.00, 241.00) | 224.50(158.75, 302.00) | <0.001 |
| NP, %, median(IQR) | 62.00(47.30, 72.50) | 72.30(61.20, 80.40) | <0.001 |
| LP, %, median(IQR) | 24.70(16.80, 38.10) | 16.90(10.78, 25.22) | <0.001 |
| MP, %, median(IQR) | 8.60(6.00, 11.20) | 8.15(6.00, 10.40) | .22 |
| EP, %, median( IQR) | 0.70(0.20, 2.00) | 0.80(0.20, 1.90) | .83 |
| BP, %, median( IQR) | 0.40(0.20, 0.60) | 0.30(0.20, 0.40) | <0.001 |
| NC, 10e9/L, median(IQR) | 3.10(2.20, 4.90) | 4.80(3.20, 7.90) | <0.001 |
| LC, 10e9/L, median(IQR) | 1.41(0.88, 1.91) | 1.20(0.80, 1.65) | 0.001 |
| MC, 10e9/L, median( IQR) | 0.46(0.32, 0.68) | 0.55(0.39, 0.83) | <0.001 |
| EC, 10e9/L, median(IQR) | 0.04(0.01, 0.12) | 0.06(0.01, 0.13) | .17 |
| BC, 10e9/L, median(IQR) | 0.02(0.01, 0.03) | 0.02(0.01, 0.03) | .33 |
| BG,mmol/l, median(IQR) | 4.92(4.50, 5.62) | 5.00(4.37, 5.91) | .58 |
| Albumin,g/L, median(IQR) | 37.40(34.40, 40.30) | 34.10(30.50, 37.90) | <0.001 |
| Cr, umol/l, median(IQR) | 67.00(55.00, 82.00) | 66.00(54.00, 83.00) | .71 |
| d-dimer,ug/l,median( IQR) | 1171.00(500.00, 2393.25) | 1387.50(710.25, 3151.50) | .02 |
| APTT,s, median (IQR) | 29.50(25.60, 33.60) | 30.50(27.80, 34.30) | .004 |
| Fibrinogen,g/l,median( IQR) | 3.28 (2.45, 4.13) | 4.86(3.50, 5.70) | <0.001 |
| CPR,mg/l, median (IQR) | 13.70(3.10, 33.94) | 49.00(19.20, 94.40) | <0.001 |
| PCT,ng/ml, median(IQR) | 0.10(0.04, 0.24) | 0.16(0.07, 0.52) | <0.001 |
| "NLR (median (IQR) ) " | 2.56(1.26, 4.06) | 4.33(2.44, 7.39) | <0.001 |
| Comorbidities, n (%) |  |  |  |
| 0 | 143(69.8) | 232(50.9) | <0.001 |
| 1 | 45(22.0) | 124(27.2) |  |
| 2+ | 17(8.3) | 100(21.9) |  |
| Hypertension, n, (%) | 31(15.1) | 160(35.1) | <0.001 |
| Diabetes, n, (%) | 7(3.4) | 67(14.7) | <0.001 |
| Cardiopathy, n, (%) | 10(4.9) | 30(6.6) | .50 |
| Nephropathy, n,(%) | 10(4.9) | 30(6.6) | .50 |
| Hepatitis B, n,(%) | 9(4.4) | 20(4.4) | 1.000 |
| Malignancy, n,(%) | 4(2.0) | 22(4.8) | .12 |

Ventricular rate(VR), Respiratory rate(RR), Ear temperature(ET), Diastolic pressure(DP), Systolic pressure(SP), hemoglobin(HB), White blood cell(WBC), Neutrophils Proportion (NP), Lymphocytes Proportion (LP), Monocytes Proportion (MP), Eosinophils Proportion (EP), Basophils Proportion (BP),Neutrophil Count(NC), Lymphocyte Count (LC), Monocyte count (MC), Eosinophil count(EC), Basophil Count(BC), Blood Glucose(BG), Creatinine(CR), activated partial thromboplastin time(APTT), Second(s), C reactive protein(CRP), procalcitonin(PCT)
