# Supplementary material for: Clinical Timing-Sequence Warning Models for Serious Bacterial Infections in Adults Based on Machine Learning: Retrospective Study
Source: J Med Internet Res. 2023 Dec 18;25:e45515. doi: 10.2196/45515 (PMC10758945; doi:10.2196/45515)
Supplement: Multimedia Appendix 3 [file jmir_v25i1e45515_app3.doc]

**Supplementary Table 2 Etiology Distribution of 945 Patients with Fever**

| **Classification of fever** | **n** |
| --- | --- |
| **Infective fever** | **945** |
| **Classification by pathogen** |  |
| Bacterial | 717 |
| Viral | 185 |
| Fungal | 17 |
| Parasite | 11 |
| Mixed infection | 4 |
| Unknown | 11 |
| **Classification by system** |  |
| Respiratory | 224 |
| Urinary | 83 |
| Digestive | 78 |
| Central nervous | 75 |
| Skin and soft tissue | 47 |
| Circulatory | 33 |
| Genital | 2 |
| Multi-system | 21 |
| Pelvic cavity or abdominal cavity | 5 |
| Unknown | 377 |
| **Non-Infective fever** | 433 |
| Autoimmune Disease | 202 |
| Neoplasm | 89 |
| Auto inflammatory diseases | 67 |
| Else | 47 |
| Unknown | 29 |
| **Unknown** | 205 |
