# Supplementary figures and images for: Clinical Timing-Sequence Warning Models for Serious Bacterial Infections in Adults Based on Machine Learning: Retrospective Study
Source: J Med Internet Res. 2023 Dec 18;25:e45515. doi: 10.2196/45515 (PMC10758945; doi:10.2196/45515)

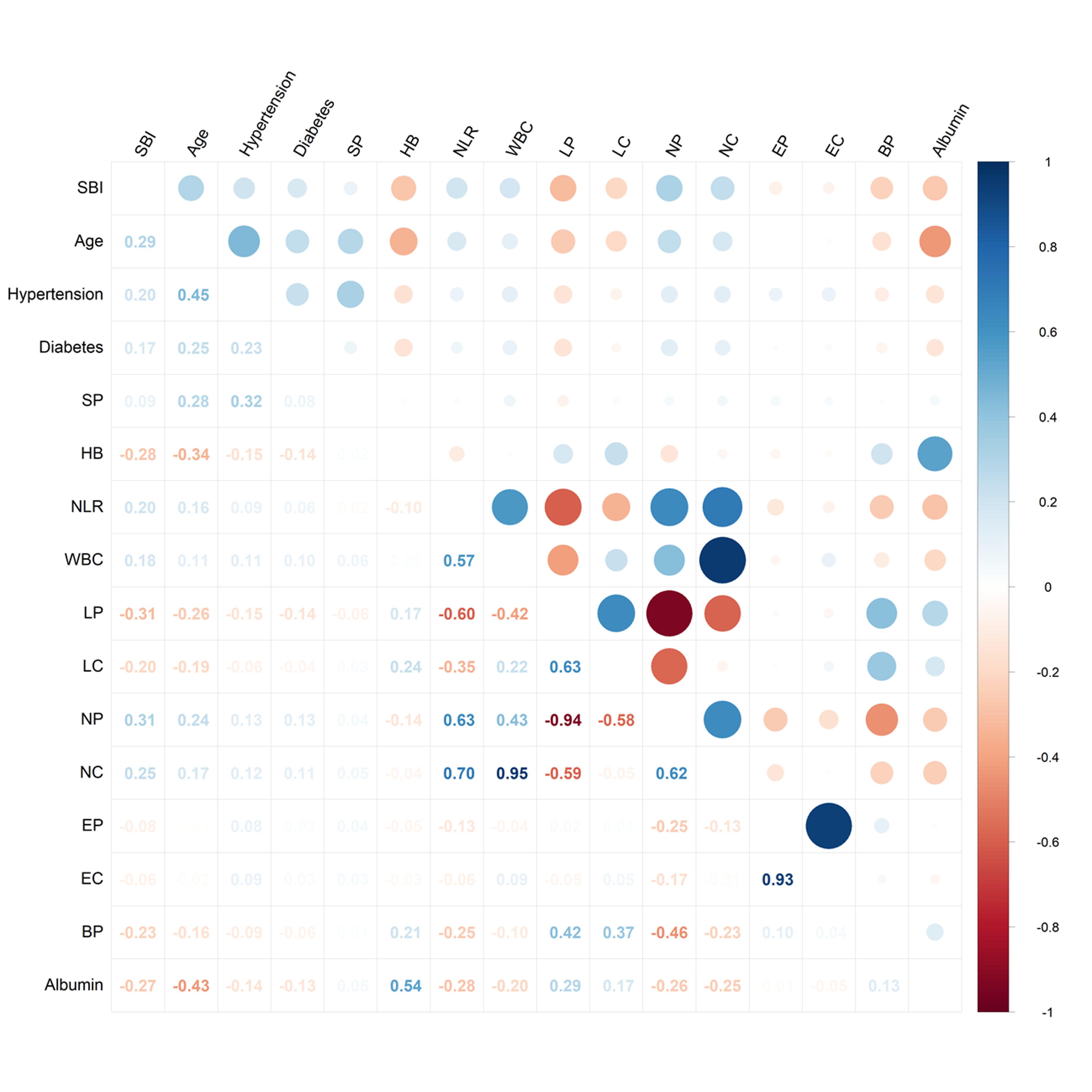

Supplement: Multimedia Appendix 4 [file jmir_v25i1e45515_app4.png]

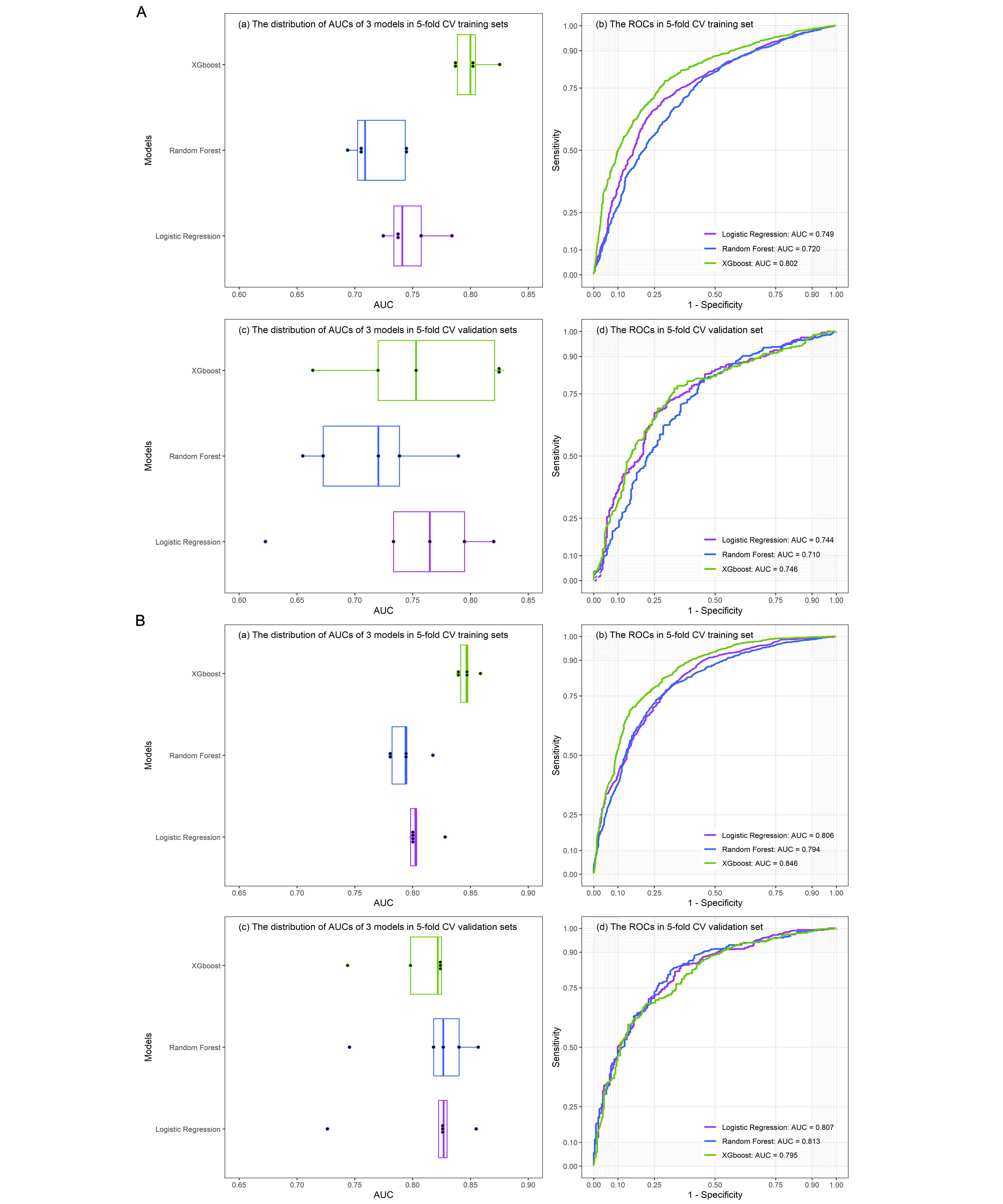

Supplement: Multimedia Appendix 5 [file jmir_v25i1e45515_app5.png]
